# Hybrid Assistive Limb Single Joint Type (HAL-SJ) Training for Knee Osteoarthritis: A Case Series

**DOI:** 10.7759/cureus.102019

**Published:** 2026-01-21

**Authors:** Mao Tanaka, Yuichiro Soma, Yukiyo Shimizu, Naoya Kikuchi, Hideki Kadone, Masashi Yamazaki, Hajime Mishima, Yasushi Hada

**Affiliations:** 1 Graduate School of Comprehensive Human Sciences, Master’s Program in Medical Sciences, University of Tsukuba, Tsukuba, JPN; 2 Department of Orthopaedic Surgery, Institute of Medicine, University of Tsukuba, Tsukuba, JPN; 3 Department of Rehabilitation Medicine, Institute of Medicine, University of Tsukuba, Tsukuba, JPN; 4 Center for Cybernics Research, University of Tsukuba Hospital, Tsukuba, JPN

**Keywords:** conservative therapy, hybrid assistive limb (hal), knee osteoarthritis/ koa, rehabilitation protocol, robotic-assisted rehabilitation

## Abstract

Knee osteoarthritis (OA) is a common degenerative joint condition in older adults, and pain often limits engagement in conventional exercise therapy. We report three cases of patients with Kellgren-Lawrence (KL) grade 3 KO who underwent rehabilitation using the Hybrid Assistive Limb Single Joint Type (HAL-SJ), a wearable robotic device that supports voluntary knee movement based on bioelectrical signals from muscle activity. All patients completed 10 training sessions over five weeks with no serious adverse events. The intervention was well tolerated, and no clinically significant deterioration in symptoms was observed during the training period. Knee pain decreased in two cases and remained unchanged in one case. These cases suggest that HAL-assisted knee training is feasible and safe for patients with moderate knee OA and may offer potential clinical benefits.

## Introduction

Knee osteoarthritis (OA) is a chronic and progressive degenerative condition that affects the entire joint complex. This disease can result in pain, reduced range of motion, and functional limitations, which can significantly diminish a person's quality of life (QOL) [1]. The Kellgren-Lawrence (KL) grading system is a widely used radiographic classification for knee OA, ranging from grade 0 (no radiographic features) to grade 4 (severe joint space narrowing with large osteophytes). Among the varying severity grades of OA, patients with moderate knee OA, categorized as KL grade 3, typically experience pronounced symptoms due to evident joint space narrowing and the presence of multiple osteophytes [2]. While conservative treatment remains the cornerstone of managing knee OA, current guidelines highlight exercise therapy as an essential component of treatment [3].

Research supports the efficacy of exercise in alleviating pain and enhancing function for individuals with this condition [4]. Exercise programs typically encompass aerobic training, strength-building activities, and balance exercises, with low-intensity options particularly suitable for patients with KL grades 2-3 [5,6]. It is advisable to adopt a multimodal approach that effectively combines exercise, weight management, pharmacological treatment, and education, all tailored by a collaborative multidisciplinary team [7]. Despite the substantial evidence underscoring the numerous benefits of exercise, many older adults face challenges in fully committing to these programs.

This reluctance can often be attributed to various psychological and social barriers, including chronic pain, reduced self-efficacy, and a general apprehension towards movement [8]. Fear-avoidance theories suggest that the fear of pain leads to avoiding activities, which results in deconditioning, functional decline, and a lower QOL [9,10]. Observational studies confirm that pain-related fear correlates with decreased physical activity and function, especially in OA patients [11]. Similarly, Japanese studies report fears of movement and uncertainty regarding suitable exercises [12]. These findings underscore the need for interventions that reduce pain and provide safe, enjoyable, and accessible exercise options.

The Hybrid Assistive Limb Single Joint Type (HAL-SJ) is a wearable robotic device that detects voluntary muscle activity through surface electromyography. It delivers proportional assistive torque to facilitate joint movements. Specifically designed to assist with the flexion and extension of a single joint, such as the knee, the HAL-SJ employs a combination of voluntary and autonomous control modes [13]. Research has demonstrated that HAL technology is effective in neurological rehabilitation, resulting in improvements in gait speed, endurance, and neuromuscular coordination in patients with conditions such as stroke [14] and spinal cord injury [13].

In Japan, HAL has been approved for insurance coverage specifically for patients with neuromuscular diseases; however, its use in the conservative management of degenerative joint diseases remains limited. Previous studies using the HAL-SJ in patients after total knee arthroplasty have reported improved early knee extension angles [15]. It remains unclear, however, whether the HAL-SJ can effectively support conservative treatment for knee OA patients who experience difficulties with conventional exercise regimens. To our knowledge, this is the first report to systematically describe the session-by-session optimization of HAL-SJ training parameters in patients with KL grade 3 knee OA managed conservatively.

This case series outlines our initial experience with the HAL-SJ in three patients diagnosed with KL grade 3 knee OA. Although these cases are part of a larger ongoing clinical trial, they were specifically selected and analyzed during an exploratory phase aimed at clarifying and optimizing the HAL training protocol for knee OA rehabilitation. The primary goal was to establish safe and effective device settings and training parameters that could be standardized for the larger trial that will follow. We hypothesized that systematically adjusting the HAL settings would enable patients to engage in therapeutic exercises with less pain and improved movement patterns, ultimately contributing to an evidence-based protocol for clinical implementation.

## Case presentation

Between July and October 2024, three patients with radiographically confirmed knee OA were enrolled. Inclusion criteria were as follows: (1) radiographic diagnosis of knee OA, (2) ability to ambulate independently without assistive devices, (3) ongoing conservative management, and (4) ability to provide written informed consent. Exclusion criteria included planned knee surgery during the intervention period, inflammatory arthropathies, uncontrolled cardiopulmonary or neurological disorders, and dermatological conditions at potential electrode attachment sites. Written informed consent was obtained from all patients before participation. This case report was conducted in the rehabilitation department of the University of Tsukuba Hospital. All procedures were performed in accordance with the Declaration of Helsinki and were approved by the institutional review board of the University of Tsukuba (approval number: TCRB24-001).

**Figure 1:**
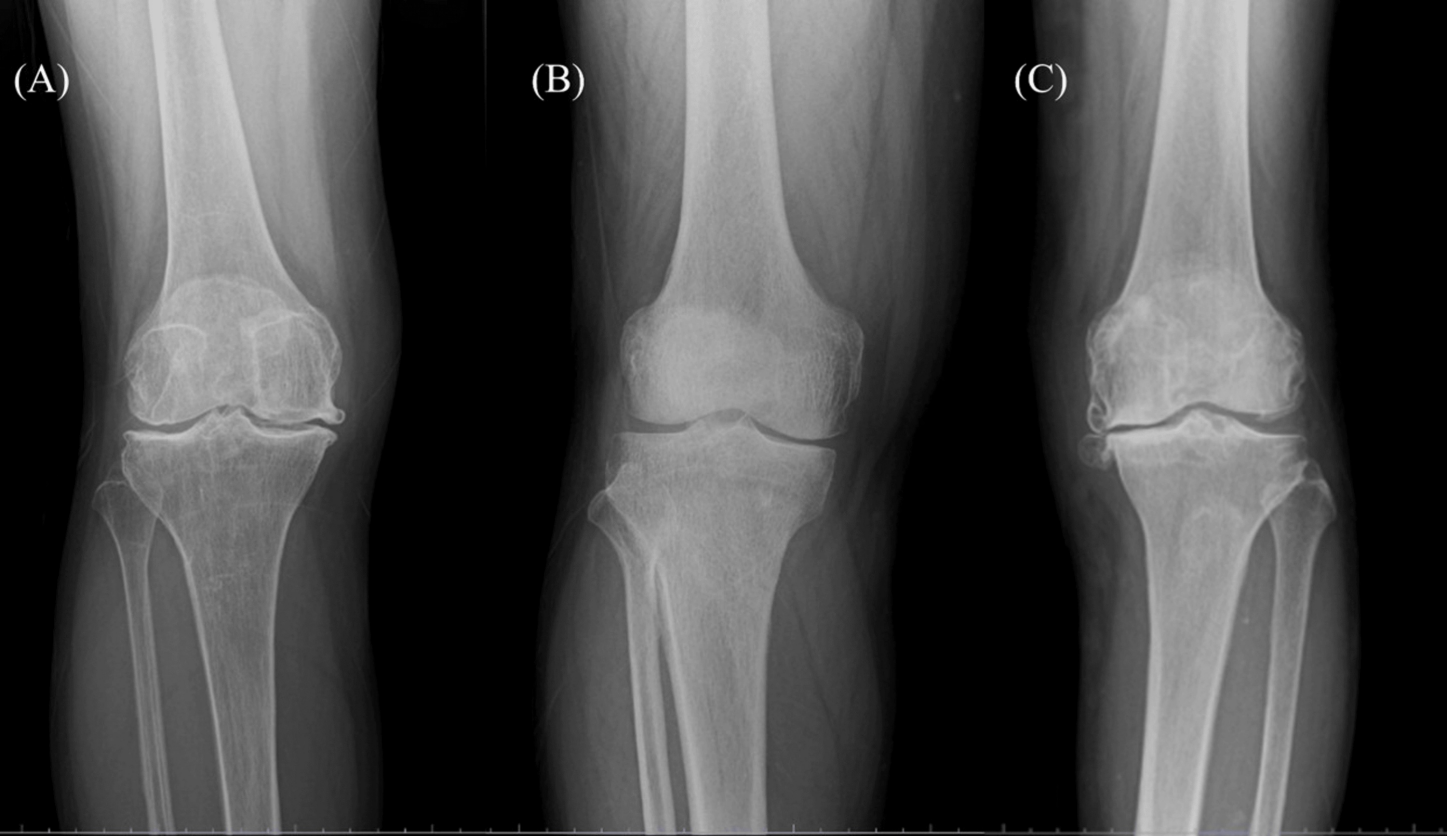
Frontal knee radiographs of the three cases demonstrating Kellgren-Lawrence grade 3 knee osteoarthritis (A) Case 1, (B) Case 2, and (C) Case 3

This case series was designed as an exploratory, single-center investigation. Because HAL-SJ training required regular hospital visits over a fixed intervention period, the number of eligible participants was limited by logistical feasibility. The reported cases represent consecutive patients who met all inclusion and exclusion criteria, consented to participate, and were able to complete the full training protocol. No patients were selected based on symptom severity, functional status, or anticipated responsiveness to HAL-SJ training. Three patients (two females and one male) with KL grade 3 knee OA completed all 10 HAL-SJ training sessions. Baseline characteristics are summarized in Table 1, and frontal knee radiographs for each case are shown in Figures 1A-1C.

**Table 1:** Baseline patient characteristics BMI: body mass index

Characteristics	Case 1	Case 2	Case 3
Age (years)	71	75	72
Sex	Female	Male	Femele
Height (cm)	155.0	159.8	157.0
Weight (kg)	50.3	76.6	61.0
BMI (kg/m^2^)	20.94	30.0	24.75

The HAL training protocol consisted of three standardized components with predefined parameter settings. During the intervention period, changes in HAL parameters were systematically recorded in all cases. Assistive torque generated by the HAL-SJ was based on bioelectrical signals derived from voluntary muscle activity. Parameter adjustments were performed by certified physical therapists at each session to ensure safe, comfortable, and effective knee movement, considering patient tolerance, movement quality, and the stability of detected bioelectrical signals. Recorded parameters included signal gain, assist level, balance between flexion and extension bioelectrical signals, torque limiter settings, and joint angle ranges for knee flexion and extension. Gain and assist levels were gradually modified across sessions to accommodate individual adaptation to HAL-assisted movement, whereas torque limiter and joint angle range settings were maintained within conservative limits to avoid excessive joint loading. Balance settings were adjusted to emphasize either knee extension or flexion, depending on the specific exercise task.

**Figure 2:**
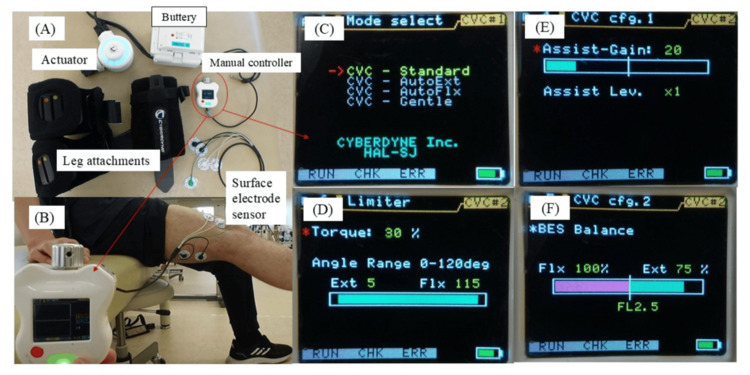
Overview of the HAL-SJ system, bioelectrical signal processing, and control interface (A) Components of the HAL-SJ, including the actuator unit, thigh and shank attachments, buttery, manual controller, and surface electrode sensors used to detect bioelectrical signals. (B) Bioelectrical potential detection and simulation before HAL-SJ, illustrating the acquisition of surface electromyographic signals and their conversion into assistive commands for joint movement. (C) Mode selection, showing the selected control mode for each exercise task, determining whether assistance was primarily driven by bioelectrical signals or by predefined motion support. (D) Torque limit setting, indicating the maximum assistive torque permitted during the task, expressed as a percentage of the device’s predefined safety threshold. (E) Assist gain setting, representing the relative magnitude of robotic assistance provided in response to the patient’s voluntary muscle activation. (F) BES balance, reflecting the weighting between biological signal-driven voluntary control and predefined motion support within the selected control mode HAL-SJ: Hybrid Assistive Limb Single Joint Type

The main components of the HAL-SJ system are shown in Figure 2A. Before each training session, surface electrodes were attached to the skin over the relevant muscles to detect bioelectrical signals corresponding to voluntary muscle activation. These signals were processed and converted into assistive commands to support knee joint motion, as schematically illustrated in Figure 2B. This bioelectrical signal-driven control allows the device to assist movement in synchrony with the patient’s intended action. Training was performed using predefined control modes selectable via the HAL-SJ interface (Figure 2C). The selected mode determined the relative contribution of biological signal-driven control and predefined motion support for each exercise task. Several adjustable parameters were configured individually for each session.

The torque limit was set to define the maximum assistive torque permitted during training and was expressed as a percentage of the device’s predefined safety threshold (Figure 2D). The assist gain level represented the relative magnitude of robotic assistance provided in response to the patient’s voluntary muscle activation (Figure 2E). In addition, the BES balance parameter was adjusted to regulate the weighting between biological signal-driven voluntary control and predefined motion support within the selected control mode (Figure 2F). These parameters were modified progressively across sessions according to patient tolerance, perceived exertion, and therapist judgment, to ensure safe assistance while encouraging active voluntary movement.

**Figure 3:**
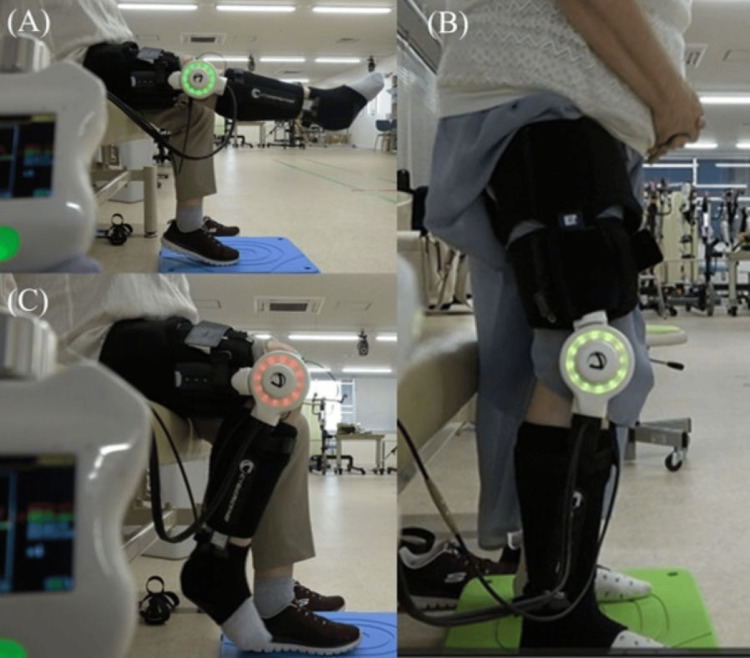
Representative HAL-SJ-assisted training tasks performed during the intervention period (A) Seated knee extension training using Auto Flexion mode. (B) Sit-to-stand training performed in Standard mode. (C) Seated knee flexion training using Auto Extension mode HAL-SJ: Hybrid Assistive Limb Single Joint Type

For knee extension training, the Auto Flexion mode was used, with the maximum extension angle limited to within 3° of each patient’s baseline active knee extension. Balance settings favored extension (extension 100%, flexion 30-50%), and patients performed two to three sets of 10-20 repetitions from a seated position (Figure 3A). Sit-to-stand training was conducted in Standard mode, initiated from an edge-of-bed sitting position with the hips and knees flexed to approximately 90°. Patients performed 10 repetitions per set (Figure 3B). For knee flexion training, the Auto Extension mode was employed, with balance settings favoring flexion (extension 30-50%, flexion 100%). Patients completed two to three sets of 10-20 repetitions (Figure 3C).

Session structure and safety monitoring training were initiated in a seated position, with the seat height adjusted to prevent the feet from contacting the floor. Each session followed a standardized sequence. First, knee extension exercises were performed from an edge-of-bed sitting position, consisting of 20 repetitions per set over approximately 10 minutes. Next, sit-to-stand practice was performed for approximately five minutes, with 10 repetitions per set. Finally, knee flexion exercises were conducted from a seated position with the knee initially held in extension, consisting of 10 repetitions per set over approximately five minutes. Rest periods of 30 seconds to one minute were provided between sets, during which patient fatigue and knee pain were assessed. Total session duration was approximately 50 minutes, including preparation, device fitting, and parameter adjustment. After each session, vital signs were reassessed, and knee pain and perceived exertion were evaluated using the visual analog scale [16] and the modified Borg scale [17], respectively.

Knee pain intensity and perceived exertion were assessed repeatedly to capture session-by-session responses to HAL-SJ-assisted training. Knee pain was evaluated using a visual analog scale (VAS; 0-100 mm) before and immediately after each HAL-SJ training session. Pre-session VAS reflected knee pain at rest before training, whereas post-session VAS reflected knee pain following completion of the session. Perceived exertion was assessed using the modified Borg scale (0-10) immediately after each training session, representing the overall subjective effort associated with that session. As secondary outcome measures, the Western Ontario and McMaster Universities Osteoarthritis Index (WOMAC), passive range of motion (ROM), and the Knee Injury and Osteoarthritis Outcome Score (KOOS) were assessed before the first HAL session and after the 10th session. During the intervention period, patients were not instructed to perform additional home-based strengthening exercises or to make substantial changes to daily activities. Safety protocols were in place to allow immediate interruption or postponement of sessions in the event of adverse symptoms, with prompt reporting to the attending physician when necessary.

Results

All three cases completed all 10 HAL-SJ training sessions without any serious adverse events or session interruptions. No clinically significant changes in blood pressure, heart rate, or peripheral oxygen saturation were observed before or after the training sessions. Minor adverse events, including transient muscle soreness and post-exercise fatigue, were reported; however, these symptoms resolved spontaneously within 24 hours and did not interfere with continued training. Session adherence was 100%, and all cases completed the intervention within the planned five-week period.

Case 1 showed a gradual reduction in assist gain level from 30% at session 1 to 10% by sessions 6-10, reflecting progressive adaptation over the intervention period (Tables 2-4). BES flexion and extension balance parameters remained largely stable across sessions, with flexion balance consistently set at 80-100% and extension balance at 100% in most sessions. Torque limiter settings were maintained within a moderate range (60%-80%), with no abrupt changes observed. After session 5, only minor adjustments in assist gain and joint angle ranges were required, indicating stabilization of neuromuscular control.

Case 2 demonstrated greater variability in parameter settings compared with Case 1, particularly during the early and middle sessions (Tables 5-7). Assist gain levels were increased from 15% at session 1 to 25% by session 2 and were largely maintained thereafter, with a slight reduction to 20% at the final session. BES flexion balance showed stepwise adjustments, increasing from 20-30% in the early sessions to 70% from session 4 onward, whereas BES extension balance remained consistently high at 100% throughout the intervention. Torque limiter settings were relatively high in the first half of the intervention (80%-90%) and were reduced to 70% in the final session, reflecting gradual optimization for safety and performance. Although assist gain levels stabilized after the initial sessions, continued modifications in balance parameters and joint angle ranges suggest ongoing individual adaptation rather than early neuromuscular stabilization.

Case 3 exhibited rapid stabilization of parameter settings after the early sessions, indicating early adaptation of neuromuscular control (Tables 8-10). Assist gain levels initially ranged from 25% to 30% during sessions 1 and 2 but were progressively reduced to 20% by session 3 and further decreased to 5% by the final session. BES flexion balance remained consistently high (approximately 100%) throughout the intervention, whereas BES extension balance showed a marked reduction after session 3, stabilizing at low levels thereafter. Torque limiter settings were gradually lowered from 90% in the initial sessions to 60%-70% in later sessions, accompanied by minor adjustments in joint angle ranges. After session 3, only minimal modifications in assist gain, balance parameters, and angle settings were required, supporting early stabilization of the neuromuscular response compared with the other cases.

**Table 2:** Setup mode of the extension exercise using Hybrid Assistive Limb in case 1 Gain: amplifies the strength of the voluntary input signal. BES balance: adjusts the relative weighting between flexion and extension signals. torque limit: sets a safety ceiling for the maximum torque output. Angle range: defines the allowable joint motion limits in flexion and extension. Count: records the number of task repetitions

Session	Assist Gain Level	BES Flexion Balance	BES Extension Balance	Torque Limit (%)	Flexion Angle Range (°)	Extension Angle Range (°)	Count
1	30	80	100	70	100	5	112
2	25	80	100	80	100	5	257
3	25	80	100	80	100	5	400
4	15	80	100	80	100	3	340
5	15	80	100	80	100	2	440
6	10	80	100	80	100	1	380
7	15	80	100	80	100	1	320
8	10	80	100	80	100	1	420
9	10	80	100	80	100	1	400
10	10	80	100	80	100	1	360

**Table 3:** Setup mode of the sit-to-stand exercise using Hybrid Assistive Limb in case 1 Gain: amplifies the strength of the voluntary input signal. BES balance: adjusts the relative weighting between flexion and extension signals. torque limit: sets a safety ceiling for the maximum torque output. Angle range: defines the allowable joint motion limits in flexion and extension. Count: records the number of task repetitions

Session	Assist Gain Level	BES Flexion Balance	BES Extension Balance	Torque Limit (%)	Flexion Angle Range (°)	Extension Angle Range (°)	Count
1	20	100	100	70	120	5	50
2	20	100	100	70	120	5	70
3	20	100	100	70	120	5	80
4	20	100	100	60	120	5	80
5	20	100	100	60	120	5	100
6	15	100	100	60	120	1	100
7	15	100	80	60	120	1	100
8	15	100	80	60	120	1	140
9	15	100	80	60	120	1	100
10	15	100	80	60	120	1	120

**Table 4:** Setup mode of flexion exercises using Hybrid Assistive Limb in case 1 Gain: amplifies the strength of the voluntary input signal. BES balance: adjusts the relative weighting between flexion and extension signals. torque limit: sets a safety ceiling for the maximum torque output. Angle range: defines the allowable joint motion limits in flexion and extension. Count: records the number of task repetitions

Session	Assist Gain Level	BES Flexion Balance	BES Extension Balance	Torque Limit (%)	Flexion Angle Range (°)	Extension Angle Range (°)	Count
1	15	100	40	70	120	5	67
2	15	100	10	70	120	20	120
3	15	100	10	70	120	30	100
4	10	100	10	70	120	30	120
5	10	100	10	70	120	30	220
6	10	100	10	70	120	30	240
7	10	100	10	80	120	30	200
8	10	100	10	70	120	30	180
9	10	100	10	70	120	40	180
10	10	100	10	70	120	40	257

**Table 5:** Setup mode of the extension exercise using Hybrid Assistive Limb in case 2 Gain: amplifies the strength of the voluntary input signal. BES balance: adjusts the relative weighting between flexion and extension signals. torque limit: sets a safety ceiling for the maximum torque output. Angle range: defines the allowable joint motion limits in flexion and extension. Count: records the number of task repetitions

Session	Assist Gain Level	BES Flexion Balance	BES Extension Balance	Torque Limit (%)	Flexion Angle Range (°)	Extension Angle Range (°)	Count
1	15	20	100	80	100	-9	280
2	25	30	100	90	100	-8	280
3	25	30	100	90	100	-7	320
4	25	70	100	90	100	-7	340
5	25	70	100	90	100	-5	360
6	25	70	100	90	100	-3	300
7	25	70	100	90	100	-3	360
8	25	70	100	90	100	-3	440
9	25	70	100	90	100	-3	340
10	20	70	100	70	100	-3	280

**Table 6:** Setup mode of the sit-to-stand exercise using Hybrid Assistive Limb in case 2 Gain: amplifies the strength of the voluntary input signal. BES balance: adjusts the relative weighting between flexion and extension signals. torque limit: sets a safety ceiling for the maximum torque output. Angle range: defines the allowable joint motion limits in flexion and extension. Count: records the number of task repetitions

Session	Assist Gain Level	BES Flexion Balance	BES Extension Balance	Torque Limit (%)	Flexion Angle Range (°)	Extension Angle Range (°)	Count
1	100	20	100	20	120	-6	60
2	100	20	100	20	120	-6	80
3	100	20	100	20	120	-5	80
4	100	20	100	20	120	-5	80
5	100	20	100	20	120	-5	80
6	100	20	100	20	120	-5	80
7	100	20	100	20	120	-5	80
8	100	10	100	30	120	-5	100
9	100	30	100	20	115	-3	100
10	100	20	100	30	115	-3	100

**Table 7:** Setup mode of flexion exercises using Hybrid Assistive Limb in case 2 Gain: amplifies the strength of the voluntary input signal. BES balance: adjusts the relative weighting between flexion and extension signals. torque limit: sets a safety ceiling for the maximum torque output. Angle range: defines the allowable joint motion limits in flexion and extension. Count: records the number of task repetitions

Session	Assist Gain Level	BES Flexion Balance	BES Extension Balance	Torque Limit (%)	Flexion Angle Range (°)	Extension Angle Range (°)	Count
1	15	100	5	80	120	40	200
2	15	100	5	80	120	40	200
3	15	100	5	80	120	40	120
4	15	100	5	80	120	40	140
5	15	100	5	80	120	40	180
6	15	100	5	80	120	40	200
7	15	100	5	80	120	40	100
8	15	100	5	80	120	40	220
9	15	100	5	80	120	40	200
10	15	100	5	80	120	40	200

**Table 8:** Setup mode of the extension exercise using Hybrid Assistive Limb in case 3 Gain: amplifies the strength of the voluntary input signal. BES balance: adjusts the relative weighting between flexion and extension signals. torque limit: sets a safety ceiling for the maximum torque output. Angle range: defines the allowable joint motion limits in flexion and extension. Count: records the number of task repetitions

Session	Assist Gain Level	BES Flexion Balance	BES Extension Balance	Torque Limit (%)	Flexion Angle Range (°)	Extension Angle Range (°)	Count
1	25	75	100	90	-3	115	220
2	30	75	100	90	-3	115	100
3	20	10	100	70	-3	115	180
4	20	10	100	70	-2	118	160
5	15	10	100	70	-2	118	160
6	15	10	100	70	-1	118	160
7	10	10	100	70	-1	119	160
8	10	10	100	60	-1	119	160
9	10	10	100	60	-1	119	160
10	5	10	100	60	0	120	160

**Table 9:** Setup mode of the sit-to-stand exercise using Hybrid Assistive Limb in case 3 Gain: amplifies the strength of the voluntary input signal. BES balance: adjusts the relative weighting between flexion and extension signals. torque limit: sets a safety ceiling for the maximum torque output. Angle range: defines the allowable joint motion limits in flexion and extension. Count: records the number of task repetitions

Session	Assist Gain Level	BES Flexion Balance	BES Extension Balance	Torque Limit (%)	Flexion Angle Range (°)	Extension Angle Range (°)	Count
1	10	70	100	80	-3	115	80
2	10	75	100	90	-3	115	80
3	10	100	100	70	-3	120	80
4	5	100	100	70	-2	118	80
5	5	100	100	70	-1	119	80
6	5	100	100	70	-1	119	80
7	5	100	100	70	-1	119	80
8	5	100	100	70	-1	119	80
9	5	100	100	60	-1	119	80
10	5	100	100	60	0	120	80

**Table 10:** Setup mode of flexion exercises using Hybrid Assistive Limb in case 3 Gain: amplifies the strength of the voluntary input signal. BES balance: adjusts the relative weighting between flexion and extension signals. torque limit: sets a safety ceiling for the maximum torque output. Angle range: defines the allowable joint motion limits in flexion and extension. Count: records the number of task repetitions

Session	Assist Gain Level	BES Flexion Balance	BES Extension Balance	Torque Limit (%)	Flexion Angle Range (°)	Extension Angle Range (°)	Count
1	15	100	95	100	115	30	140
2	15	100	95	100	115	30	140
3	15	100	40	80	120	20	140
4	15	100	30	70	120	30	100
5	15	100	25	70	120	30	140
6	10	100	25	70	120	30	140
7	10	100	20	70	120	40	160
8	10	100	20	70	120	40	160
9	5	100	20	60	120	40	140
10	5	100	20	60	120	40	140

Session-by-session changes in knee pain and perceived exertion are presented in Table 11. Knee pain intensity, assessed using the VAS before and after each training session, showed case-specific patterns across the intervention period. In Case 1, post-session VAS values were frequently lower than pre-session values, suggesting a transient reduction in knee pain following HAL-assisted training. Case 2 demonstrated greater variability in both pre- and post-session VAS scores, with several sessions showing higher baseline pain levels, whereas Case 3 exhibited relatively stable pain levels with minimal within-session fluctuation. Perceived exertion, assessed using the modified Borg scale before and after each session, remained consistently low across all cases. Post-session Borg scores generally ranged from 0 to 4, indicating that HAL-SJ-assisted training was performed without excessive physical strain. No case demonstrated progressive increases in perceived exertion over successive sessions. Importantly, no session-related exacerbation of knee pain or excessive fatigue was observed that required interruption or discontinuation of training.

**Table 11:** Session-by-session changes in knee pain (VAS) and perceived exertion (modified Borg scale) during HAL-SJ training VAS-baseline: knee pain intensity assessed using a VAS (0-100 mm) immediately before each HAL-SJ training session. VAS-post-intervention: knee pain intensity assessed using the same scale immediately after completion of each session. Borg-baseline: perceived exertion assessed using the modified Borg scale (0-10) immediately before each session. Borg-post-intervention: perceived exertion assessed using the same scale immediately after each session VAS: visual analog scale; HAL-SJ: Hybrid Assistive Limb Single Joint Type

Case 1				Case 2				Case 3			
VAS-Baseline	VAS-Post-intervention	Borg-Baseline	Borg-Post-intervention	VAS-Baseline	VAS-Post-intervention	Borg-Baseline	Borg-Post-intervention	VAS-Baseline	VAS-Post-intervention	Borg-Baseline	Borg-Post-intervention
10	0	0.5	3	18	8	0	0	7	7	0.5	0.5
10	10	0.5	3	15	7	0	1	6	5	1	1
5	0	0.5	2	12	4	0	0	7	6	1	2
9	3	0.5	2	33	7	0	0	15	5	2	1
12	7	0.5	4	18	9	0	0	10	9	1	1
12	8	0	4	19	8	0	0	9	8	1	1
9	4	0	4	0	2	0	0	7	6	1	2
8	3	0	3	8	7	0	0	8	4	1	1
5	5	0	3	15	13	1	2	7	6	1	1
12	5	0.5	2	5	5	0	0	9	8	1	1

Individual changes in knee ROM, KOOS, and WOMAC scores before the first HAL session and after the 10th session are shown in Table 12. Knee extension ROM improved in all three cases following the intervention, with reductions in extension deficits. Knee flexion ROM also increased in all cases after HAL training. WOMAC scores decreased in Cases 1 and 3, indicating an improvement in symptom severity, whereas no change was observed in Case 2. KOOS scores showed minimal changes, with a slight increase in Case 1 and small decreases in Cases 2 and 3.

**Table 12:** Changes in knee range of motion, KOOS, and WOMAC scores before and after HAL training HAL: Hybrid Assistive Limb; WOMAC: Western Ontario and McMaster Universities Osteoarthritis Index; KOOS: Knee Injury and Osteoarthritis Outcome Score; ROM: range of motion; pre-HAL: before the first HAL session; post-HAL: after the 10th HAL session

Characteristics		Case 1	Case 2	Case 3
Extension ROM (°)	Pre-HAL	-4	-10	-7
	Post-HAL	-2	-4	-2
Flexion ROM (°)	Pre-HAL	113	119	128
	Post-HAL	122	126	140
KOOS score	Pre-HAL	63	57	90
	Post-HAL	64	53	85
WOMAC score	Pre-HAL	19	23	4
	Post-HAL	9	23	3

## Discussion

This exploratory study demonstrates the safety and feasibility of a knee training protocol using the HAL-SJ in older adults with KL grade 3 knee OA undergoing conservative management. Importantly, the study identified optimal parameter settings for HAL use in this population. To our knowledge, this is one of the first reports to systematically document and analyze session-by-session HAL-SJ parameter optimization specifically in patients with knee OA, a population for which such guidance has previously been lacking. All participants completed all 10 training sessions without serious adverse events, indicating that HAL-assisted training is a viable and well-tolerated intervention even in elderly individuals with musculoskeletal limitations.

A key contribution of this study is the detailed documentation of HAL parameter settings. Although HAL technology has demonstrated efficacy in neurological conditions, specific parameter guidelines for musculoskeletal disorders have remained unclear. Similar to the comprehensive staged protocol proposed by Ueno et al. for stroke rehabilitation, the present findings provide practical, data-driven recommendations to support clinical implementation. The detailed records presented in Tables 1-3 address an important gap in the literature and may serve as a valuable reference for future studies.

Although the overall ranges of HAL-SJ parameter settings were relatively similar across cases, clinical responses differed between individuals. This indicates that symptomatic and functional outcomes were not determined solely by the magnitude of robotic assistance. Patient-specific factors such as baseline pain intensity, BMI, functional status, and neuromuscular responsiveness likely played a significant role in shaping individual training responses. In particular, the limited improvement observed in Case 2 despite comparable parameter progression suggests that mechanical joint loading or disease-related constraints, rather than insufficient parameter adjustment, may have influenced outcomes. Importantly, this study was not designed to prioritize or formally assess clinical efficacy. Outcome measures such as pain, ROM, and patient-reported function were included solely for exploratory purposes, to observe potential trends and variability in response during HAL-SJ-assisted training.

Improvements observed in both knee extension and flexion ranges of motion, particularly the reduction in extension deficits, support the idea that HAL-based training may promote neuromuscular re-education. By assisting voluntary movement through the detection of bioelectrical signals, HAL functions not as a passive orthotic device but as an active facilitator of motor learning. This mechanism may help decrease fear-avoidant behavior, which is commonly seen in elderly individuals with knee OA. Furthermore, the minimal changes in perceived exertion (modified Borg scale ≤4) despite functional improvements suggest that HAL enables efficient movement patterns with a reduced physical burden, potentially modulating inflammatory responses associated with excessive mechanical stress. However, changes in patient-reported outcome measures were limited over the short intervention period, indicating that improvements in joint mobility may occur before measurable changes in subjective functional outcomes.

Given the increasing global burden of knee OA and the growing demand for rehabilitation services, developing effective conservative treatment strategies is critically important. Nevertheless, several limitations of this study should be acknowledged. First, the small sample size (n = 3) limits the generalizability of the findings. Second, the absence of a control group prevents definitive conclusions regarding the superiority of HAL-assisted training compared with conventional physiotherapy. Third, interindividual differences in baseline pain intensity and functional status may have contributed to variability in outcomes. In addition, BMI varied across cases, and excess body weight is a well-established factor contributing to mechanical joint overload and disease progression in knee OA; therefore, BMI may have influenced individual training responses in this small series.

Finally, the optimal frequency, intensity, and duration of HAL-assisted training remain to be determined. In addition, the development of an ideal HAL-SJ training protocol will require systematic evaluation of efficacy-related outcomes, including ROM, muscle strength, and walking performance, in future studies. Future studies are needed to identify patient phenotypes and functional profiles that gain the greatest benefit from HAL-assisted therapy. A planned randomized controlled trial will compare this optimized HAL protocol with conventional physiotherapy to establish its clinical effectiveness. Additional investigations should also explore patient selection criteria and cost-effectiveness to support broader clinical adoption. Based on the safety, feasibility, and parameter optimization data obtained from these three cases, a preliminary standardized protocol was developed to guide future large-scale clinical studies, with details provided in Appendix A.

## Conclusions

HAL-SJ was safely implemented in three patients with KL grade 3 knee OA undergoing conservative treatment, with no serious adverse events. Knee pain improved in two cases and was essentially unchanged in one. These findings support the feasibility and short-term tolerability of HAL-assisted knee training in this preliminary case series. Further studies are required to determine its clinical effectiveness and to refine patient selection and training protocols.

## Appendices

Training schedule

Ten sessions conducted over four to six weeks, performed two to three times per week with no more than seven days between sessions. Each session includes approximately 20 minutes of active training (total session duration approximately 50 minutes, including preparation).

HAL parameter settings

Knee Extension

Auto Flexion mode, with range limited to baseline active extension +3°; balance settings: extension 100/flexion 30-50

Sit-to-Stand Practice

Standard mode initiated from an edge-of-bed position (hips and knees at 90°)

Knee Flexion

Auto Extension mode; balance settings: extension 30-50/flexion 100 progression

Guidelines

Initial gain settings should begin at 20-30% and be gradually increased to 40-50% according to participant tolerance and bioelectrical signal quality. Assist levels and balance settings should be individualized based on each participant’s movement impairments.

Safety Monitoring

Pain (VAS), vital signs, and perceived exertion (modified Borg scale ≤4) should be monitored before and after each session. Training should be discontinued if VAS exceeds 80 mm or if vital signs change by more than 20% from baseline.

Implementation Requirements

All sessions must be supervised by certified physical therapists trained in HAL operation, with real-time parameter adjustments guided by bioelectrical signal feedback displayed on the controller interface. This protocol provides a standardized yet adaptable framework for the clinical application of HAL-SJ in knee OA rehabilitation and addresses the current lack of detailed parameter guidance for this population.

## References

[1] Imoto AM, Peccin MS, Trevisani VF (2012). Quadriceps strengthening exercises are effective in improving pain, function and quality of life in patients with osteoarthritis of the knee. Acta Ortop Bras.

[2] Kohn MD, Sassoon AA, Fernando ND (2016). Classifications in brief: Kellgren-Lawrence classification of osteoarthritis. Clin Orthop Relat Res.

[3] Lespasio MJ, Piuzzi NS, Husni ME, Muschler GF, Guarino A, Mont MA (2017). Knee osteoarthritis: a primer. Perm J.

[4] Arden NK, Perry TA, Bannuru RR (2021). Non-surgical management of knee osteoarthritis: comparison of ESCEO and OARSI 2019 guidelines. Nat Rev Rheumatol.

[5] Lim WB, Al-Dadah O (2022). Conservative treatment of knee osteoarthritis: a review of the literature. World J Orthop.

[6] Cheung C, Wyman JF, Bronas U, McCarthy T, Rudser K, Mathiason MA (2017). Managing knee osteoarthritis with yoga or aerobic/strengthening exercise programs in older adults: a pilot randomized controlled trial. Rheumatol Int.

[7] Bannuru RR, Osani MC, Vaysbrot EE (2019). OARSI guidelines for the non-surgical management of knee, hip, and polyarticular osteoarthritis. Osteoarthritis Cartilage.

[8] McAuley E, Szabo A, Gothe N, Olson EA (2011). Self-efficacy: implications for physical activity, function, and functional limitations in older adults. Am J Lifestyle Med.

[9] Kanavaki AM, Rushton A, Efstathiou N, Alrushud A, Klocke R, Abhishek A, Duda JL (2017). Barriers and facilitators of physical activity in knee and hip osteoarthritis: a systematic review of qualitative evidence. BMJ Open.

[10] Vlaeyen JWS, Linton SJ (2000). Fear-avoidance and its consequences in chronic musculoskeletal pain: a state of the art. Pain.

[11] Skou ST, Pedersen BK, Abbott JH, Patterson B, Barton C (2018). Physical activity and exercise therapy benefit more than just symptoms and impairments in people with hip and knee osteoarthritis. J Orthop Sports Phys Ther.

[12] Uritani D, Ikeda A, Shironoki T, Matsubata K, Mutsura Y, Fujii T, Ikeda K (2021). Perceptions, beliefs, and needs of Japanese people with knee osteoarthritis during conservative care: a qualitative study. BMC Musculoskelet Disord.

[13] Wall A, Borg J, Palmcrantz S (2015). Clinical application of the Hybrid Assistive Limb (HAL) for gait training: a systematic review. Front Syst Neurosci.

[14] Watanabe H, Tanaka N, Inuta T, Saitou H, Yanagi H (2014). Locomotion improvement using a hybrid assistive limb in recovery phase stroke patients: a randomized controlled pilot study. Arch Phys Med Rehabil.

[15] Yoshioka T, Kubota S, Sugaya H, Arai N, Hyodo K, Kanamori A, Yamazaki M (2021). Feasibility and efficacy of knee extension training using a single-joint hybrid assistive limb, versus conventional rehabilitation during the early postoperative period after total knee arthroplasty. J Rural Med.

[16] Huskisson EC (1974). Measurement of pain. Lancet.

[17] Borg G (1990). Psychophysical scaling with applications in physical work and the perception of exertion. Scand J Work Environ Health.

[18] Ueno T, Marushima A, Kawamoto H (2022). Staged treatment protocol for gait with Hybrid Assistive Limb in the acute phase of patients with stroke. Assist Technol.

[19] Kisand K, Tamm AE, Lintrop M, Tamm AO (2018). New insights into the natural course of knee osteoarthritis: early regulation of cytokines and growth factors, with emphasis on sex-dependent angiogenesis and tissue remodeling. A pilot study. Osteoarthritis Cartilage.

[20] (2023). Global, regional, and national burden of osteoarthritis, 1990-2020 and projections to 2050: a systematic analysis for the Global Burden of Disease Study 2021. Lancet Rheumatol.

[21] Cieza A, Causey K, Kamenov K, Hanson SW, Chatterji S, Vos T (2021). Global estimates of the need for rehabilitation based on the Global Burden of Disease study 2019: a systematic analysis for the Global Burden of Disease Study 2019. Lancet.

